# Distributed Temperature Sensing Monitoring of Well Completion Processes in a CO_2_ Geological Storage Demonstration Site

**DOI:** 10.3390/s18124239

**Published:** 2018-12-03

**Authors:** Dasom Sharon Lee, Kwon Gyu Park, Changhyun Lee, Sang-Jin Choi

**Affiliations:** 1 Petroleum & Marine Division, Korea Institute of Geoscience and Mineral Resources, 124 Gwahak-ro, Yuseong-gu, Daejeon 34132, Korea; dslee@kigam.re.kr (D.S.L.); sang-jin@kigam.re.kr (S.-J.C.); 2 Department of Energy System Engineering, Seoul National University, 1 Gwanak-ro, Gwanak-gu, Seoul 08826, Korea

**Keywords:** distributed temperature sensing, temperature calibration, depth correction, fiber optic sensing, well completion monitoring

## Abstract

The Distributed Temperature Sensing (DTS) profiles obtained during well completion of a CO_2_ monitoring well were analyzed to characterize each well completion process in terms of temperature anomalies. Before analysis, we corrected the depth by redistributing the discrepancy, and then explored three temperature calibration methods. Consequently, we confirmed the depth discrepancy could be well corrected with conventional error redistribution techniques. Among three temperature calibration methods, the conventional method shows the best results. However, pointwise methods using heat coil or in-well divers also showed reliable accuracy, which allows them to be alternatives when the conventional method is not affordable. The DTS data revealed that each well completion processes can be characterized by their own distinctive temperature anomaly patterns. During gravel packing, the sand progression was monitorable with clear step-like temperature change due to the thermal bridge effect of sand. The DTS data during the cementing operation, also, clearly showed the progression up of the cement slurry and the exothermic reaction associated with curing of cement. During gas lift operations, we could observe the effect of casing transition as well as typical highly oscillating thermal response to gas lifting.

## 1. Introduction

The most distinctive advantage of fiber optic Distributed Temperature Sensing (DTS) technology over other point sensing and wireline logging technologies is its ‘distributed sensing’ feature. The distributed sensing, as the fiber itself is the sensing medium, enables obtaining a temperature profile for the entire fiber length with much higher spatial density than point sensors in a period. It also enables continuous real-time temperature profiling at any time throughout the well, once it is permanently installed inside the well [1]. Unlike the production logging run, which obtains temperatures after the event, the DTS can identify in-well performance changes as the events occur during production and shut-in [2]. In addition, fiber optic distributed sensing has many more advantages compared to conventional point sensors or wireline logging as it can be deployed in any harsh or unusual environment and it is small, light, corrosion–resistant, and has a long sensing range, good sensitivity, and electromagnetic immunity. 

In May of 2017, as a part of an onshore pilot-scale CO_2_ sequestration demonstration project in Korea, multifunctional monitoring wells with discrete sensors and fiber optic cable for DTS and Distributed Acoustic Sensing (DAS) have been completed. A key consideration factor in developing injection and/or monitoring wells for CO_2_ geological storage is the integrity of the well that prevents the wells from becoming a conduit for CO_2_ leakage. Generally, many sensors and pieces of equipment are installed in CO_2_ monitoring well depending on the aim of the monitoring. Therefore, more care is needed for well design, installation, and completion process in order to ensure that the many sensors and the equipment perform at their best as well as to maintain integrity. Since we applied a permanent method using gravel packing and cementing for the completion, which is irreversible unlike a non-permanent method that uses a packer, monitoring each well completion process is critical to determine whether each process has gone as planned.

For the purpose of monitoring each well completion process in terms of temperature anomaly patterns, we monitored DTS data during whole completion processes without any intervention. However, on-site analysis was limited to qualitative interpretation because temperature pre-calibration and depth correction were not possible due to the field schedule and conditions. 

In this paper, we applied a depth correction method and three temperature post-calibration methods for more quantitative and accurate analysis. Then, we characterized the thermal characteristics of each well completion process in order to explore the feasibility of DTS as a well completion monitoring and control tool.

The uncertainty of depth accuracy is inevitable in practical application of DTS to boreholes because it is difficult to detect any depth errors due to stretching and wrapping of the cable during installation. In this paper, conventional depth correction is applied by considering both stretching and wrapping of cable [3]. Then, we confirmed the accuracy of depth correction with our gas lift temperature profile using the depth of casing transition as a priori information. 

The conventional way of temperature calibration is submerging both ends of the fiber in ice or hot water to calibrate the temperature offset to an absolute value and its rate of change along the fiber length [3]. It is commonly recommended to apply temperature calibration before installation. In many cases, however, calibration before installation is not possible or limited due to practical factors like site schedule and conditions. This was our case, therefore, we corrected temperature after installation. In addition to a conventional temperature calibration method of submerging the long length in water, we also explored the possibility of two types of pointwise post-calibration methods that may overcome the practical limitation of the submerging method on the site. 

During well completion, real-time DTS data were analyzed to monitor the entire well completion process in terms of temperature changes and to control or optimize the process. However, it was limited to a qualitative analysis because the depth correction and temperature calibration were not done. After the depth correction and temperature calibration, thermal events of each well completion process could be characterized more precisely, and each well completion process could be identified with distinctive temperature anomaly pattern. The progress up the well of sand and cement slurry were monitored with clear step-like temperature change. Thermal events due to exothermal reaction during cement curing process was also clearly observed. Consequently, we could conclude that observing the entire well completion process with pre-calibrated DTS data can be a promising tool of real-time control and optimization of well completion process.

## 2. Materials and Methods

### 2.1. Site Description

**Figure 1:**
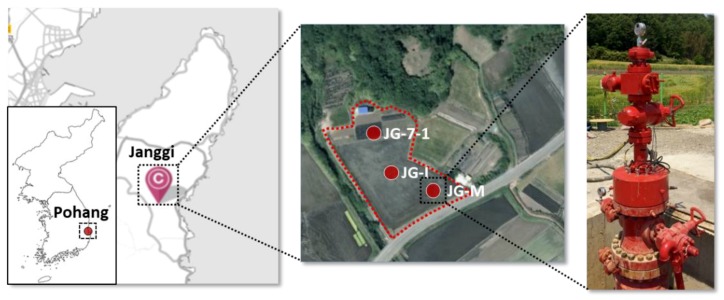
Location of Janggi field site including relative position of auxiliary monitoring well (JG-7-1), monitoring well (JG-M) with its wellhead, and injection well (JG-I).

The thermal data were collected from the monitoring well, JG-M, which was completed at the end of May 2017 as a part of onshore pilot-scale CO_2_ geological storage demonstration program at Janggi field. The Janggi field site is located at Pohang city which is the southeastern part of Korean Peninsula as illustrated in Figure 1. The total depth (TD) of the monitoring wells is 1092.44 m, and the well is slanted by 2° to the southeast direction.

### 2.2. Well Schematic and Completion Process

**Figure 2:**
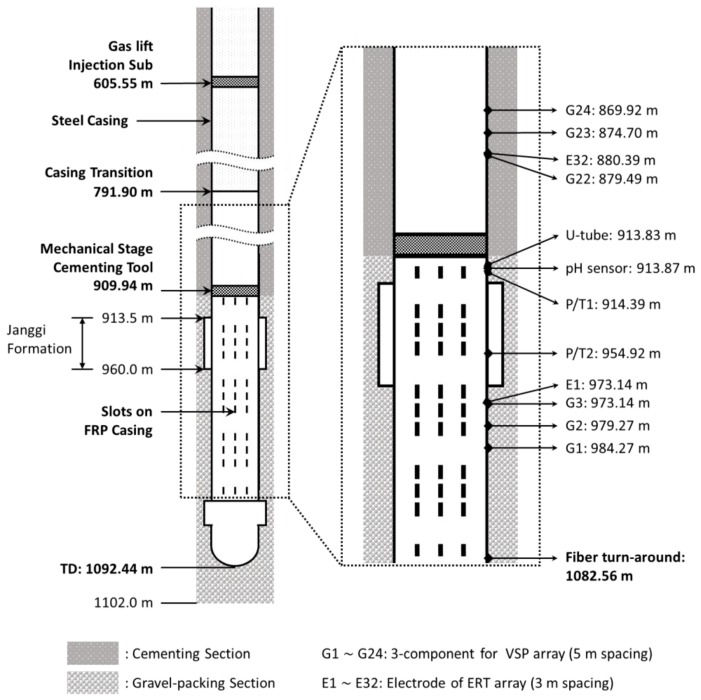
Schematic of the monitoring well, JG-M and the completion assembly employed with gravel packing and cementing.

The monitoring sensors and equipment deployed in JG-M include 32-level electrode array, 24-level 3C geophone strings, a U-tube [4] fluid sampler, two piezo pressure/temperature sensors (P/T), an experimental pH sensor, and a fiber cable for DAS, DTS, and heat-pulse monitoring [5]. The schematic of the Janggi JG-M monitoring well and the completion assembly employed is presented in Figure 2 to help understand the thermal events associated with completion, equipment and materials. 

The 32-level electrodes were installed for surface-to-borehole and cross-well Electrical Resistivity Tomography (ERT) to image the distribution and migration of the CO_2_ plume. Due to fact the 32 electrodes cover a 93 m interval around the reservoir formation (JGF = Janggi conglomerate formation), almost all materials below 803 m should be non-metallic or electrically insulated. Therefore, instead of steel casing, 3-1/2″ Fiberglass Reinforced Plastic (FRP) casing was used at 300.54 m interval below 791.90 m, where the slotted (depicted by dashed line in Figure 2) and non-slotted casing alternated to guarantee fluid circulation and to maintain strength against pressure. The 3C geophones were installed for the purpose of acquiring Vertical Seismic Profiling (VSP) and passive micro-seismic monitoring data. The P/T sensors were installed at the top and bottom boundary of JGF reservoir, which were also used for pressure and temperature during the well completion. The fiber cable turnaround which protect spliced part of fiber core for dual-ended mode measurement was installed at 1082.56 m. The gas-lift manifold for fluid production and cementing stage tool for cementing were installed at 605.55 m and 909.94 m, respectively.

All sensors and instruments are installed in the tubing by the convey method by attaching sensors and control line to 3-1/2″ steel and FRP casing. A series of centralizers and clamps were used to protect the cables during installation and cement jobs. For the FRP casing section, we used nylon decentralizers which were attached to the casing with 3/8-inch set screws. The mid joint clamp was used to hold the stainless-steel tubes. For the steel casing section, cannon cross coupling protectors with fins provide centralization during cementing. 

The two different patterns in the well schematic shown in Figure 2 depict the well completion schemes behind the casing. The bottom section below 910 m indicates the gravel packing section. The basic requirement of the reservoir section is to guarantee the circulation of pore fluid and CO_2_. The ERT and VSP sensors should be well coupled with the surrounding formations for sensing, which is the reason for choosing the gravel packing over a packer. Above 910 m of the well was completed with cementing to ensure the pressure isolation of the reservoir section and the mechanical integrity of well that prevents the well from becoming a conduit for CO_2_ migration. 

After landing the tubing hanger, DTS system and permanent pressure temperature gauge was used to monitor the process of gravel packing and cementing operation in May 2017. We directly injected 12/20 sand into the annulus and recirculated the fluid up with the 1.9-inch IJ tubing. After the gravel packing procedure, cementing is conducted by circulating slurry into the well with a stage cementing tool at 909.94 m. 

### 2.3. Fiber Optic and DTS Interrogator Configuration

**Figure 3:**
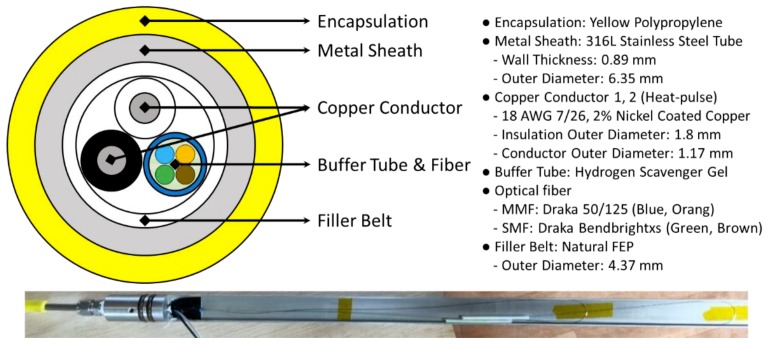
Schematic of fiber optic control line and specifications (**above**), and photo of turnaround sub for dual-ended configuration (**below**).

The fiber optic control line used in JG-M consists of four optical fiber lines for DTS and DAS and two copper conduct lines for heat-pulse monitoring [5], which are protected by stainless steel sheaths and polypropylene jackets to prevent them from being damaged during installation. Among the four optical fiber lines two single-mode fibers are for DAS and the other two are multi-mode fibers for DTS. Temperature profiles are obtained in dual-ended mode by splicing two multi-mode fibers at the end of the control line. The schematic of optical fiber control line and specification, and turnaround sub are as shown in Figure 3. 

Silixa’s XT-DTS DTS interrogation system was used to monitor the temperature changes for five days during completion and for a month during curing. The data was obtained using a passive mode where no active heating was done to the cable itself. Our interrogation unit can measure temperature profiles up to 5 km, with minimum spatial and temporal resolution of 0.25 m and 5 s, respectively. The temperature profiles were collected with a spatial sampling interval of 0.25 m, with a temporal interval of 600 s during well completion monitoring from May to June of 2017 and 60 s during background monitoring and temperature calibration tests from November, 2017 to April, 2018.

## 3. Depth and Temperature Correction

There are many potential factors that can cause errors when using the DTS system. The errors which arise from the instrumentation, the fiber, the nature of the installation, and other sources show up as depth discrepancies and temperature errors [3]. In order to specify the exact location of thermal events and to analyze the events more quantitatively, we applied depth correction and temperature calibration before interpreting thermal events for the well completion processes. 

### 3.1. Depth Correction

Depth mismatch is an unavoidable issue in the field. Unlike temperature correction, depth discrepancies cannot be pre-calibrated before installation. Even though the cables were carefully handled during the installation, many factors such as cable coiling up around the tubing, stretching of fiber optic cable itself, the usage of a decentralizer, and inner fiber optic twisting may inevitably introduce depth discrepancies. Although it may depend on the application, Smolen and van der Spek [3] stated that generally a depth mismatch of ± 0.3 m is realistic for most applications. With the depth issue comes the issue of determining the end of the fiber depth. The length measured by DTS should be mapped for depth correction to a real physical profile length of interest. In dual-ended or partially returned configurations in borehole applications, the end of the fiber can be easily identified with the peak temperature due to ambient geothermal gradient and it can be confirmed in the raw data by a sudden intensity drop around the splicing point. 

In our case, the peak temperature was observed mostly around 1197.114 m in fiber length. On the other hand, the maximum intensity drop occurred between 1196.606 m and 1196.860 m with the difference of 21.17 A.U. (relative decrease of 10.4%) and the second maximum drop occurred between 119.860 m to 1197.114 m with the difference of 18.62 A.U. (relative decrease of 9.3%). Although there is a difference of 0.5 m between the peak temperature point and the maximum intensity drop point, we used the peak temperature point as the fiber-end to determine total fiber length because such a difference is negligible considering our spatial sampling interval of 0.25 m and the width of the probe pulse. With respect to this fiber end, the fiber cable length ($D_{f}$) that corresponds to total casing length of 1082.56 m is 1095.599 m. Therefore, in our case, a depth mismatch (ΔD) of 13.039 m was identified, which is 1.2% longer than the total casing length. 

A simple way to match depth discrepancies is to redistribute the amount of depth mismatch to the total length. Thus, we redistributed total depth error throughout the total length under the assumption that the rate of depth mismatch is constant throughout the total length. 

**Figure 4:**
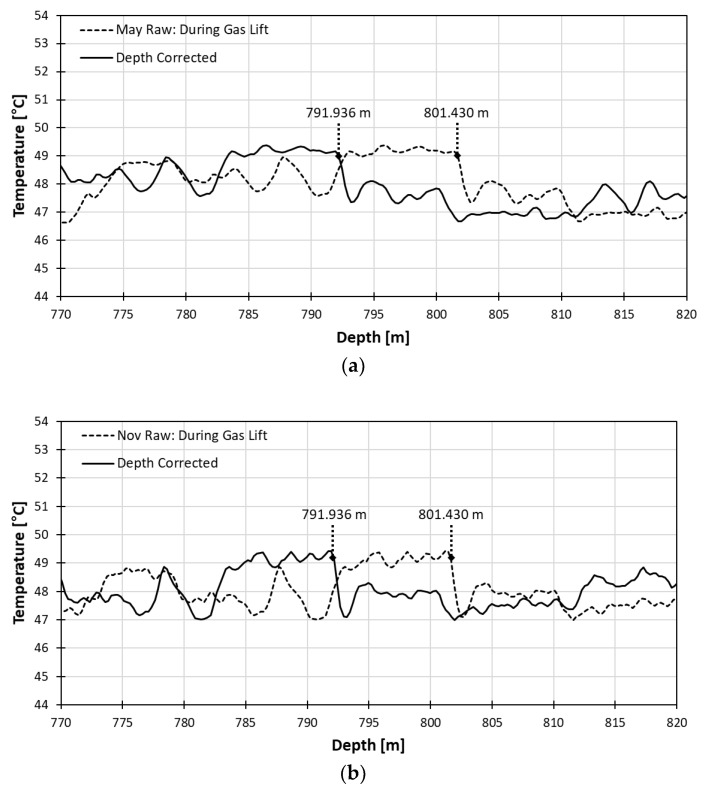
Comparison of depth correction effect with gas lift data from May (**a**) and November (**b**).

For the confirmation of depth correction accuracy, we compared the temperature profiles before and after correction for gas lift process in May and November, 2017 as shown in Figure 4. Since the casing material is changed from steel to FRP casing at 791.90 m as shown in Figure 2, any temperature anomaly associated with the heat conductivity difference of two casing material should be identified around this depth. The effect of casing transition is clearly identifiable with the temperature increase pattern from data in both May and November, regardless of depth correction. 

After correcting the depth, the depth of thermal changes due to casing transition moved up 9.494 m from 801.430 m to 791.936 m. The depth after correction, 791.936 m matches well with the true depth of the casing transition point (791.90 m) within one sampling-point difference, which is 0.25 m in terms of the DTS system’s sampling interval. In both May and November, the difference to the true depth was 0.036 m. 

Considering the true depth of casing transition point is known and the position of the thermal anomaly due to casing transition can be clearly identifiable, we may redistribute total depth error to each section above and below the transition point section by section under the assumption that the rate of depth mismatch is constant but different in each section. However, the recalculated sampling interval turns out to be the same to the third decimal place regardless of the section division. Thus, no major improvement in depth calibration using sections was seen. 

**Figure 5:**
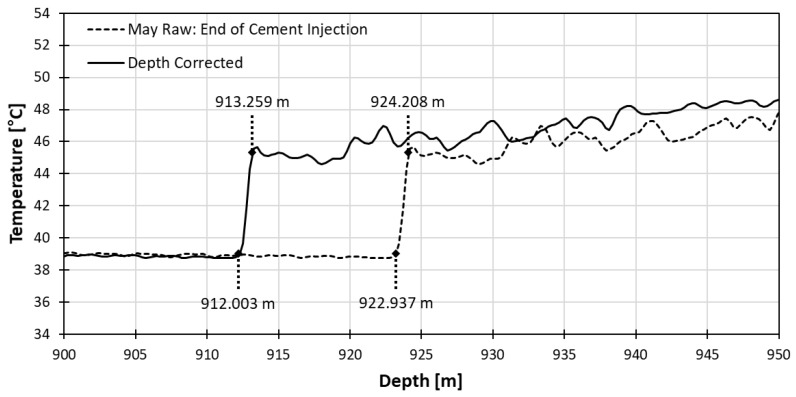
Temperature profiles before and after applying depth correction to data at the end of cement injection in May.

The new sampling interval was applied to the cement injection data for the verification of the depth correction effect again as shown in Figure 5. Although there is no known absolute position of the top of the gravel pack and the bottom of cementing, the top of the gravel pack should be near 911 m and bottom of cement injection near 910 m according to the completion schematic shown in Figure 2. 

Once the cement slurry meets the gravel pack, it will permeate slowly into the gravel pack. This process can be delineated by a temperature transition between the low temperature of the cooler cement slurry and the high temperature caused by the warmer gravel pack as shown in Figure 5, and this thermal transition remains until the temperature goes back to the normal geothermal gradient.

Considering this, we can interpret the start and the end of the thermal transition zone to the top of gravel pack and the bottom of cement slurry, respectively. The top numbers and the bottom numbers in the figure indicate the depth of the bottom of cement and the depth of the top of gravel pack, respectively. After correcting the depth, the top of gravel pack moved up 10.934 m from 922.937 m to 912.003 m that is 2.003 m difference with expected depth of 910 m. The bottom of cement moved up 10.949 m from 924.208 m to 913.259 m that is 2.259 m with expected depth of 911 m. 

### 3.2. Temperature Correction

Many DTS acquisition systems have an internal calibration system, however higher accuracy is needed for geoscience operating environments where the fiber optic cable may be under conditions of rapid change and large temperature fluctuations. Under static conditions, the accuracy of calibrated temperatures is generally within ± 0.3 °C, however under rapid operating conditions, the accuracy range becomes widened to ± 2 °C [6]. The temperature measurement accuracy also declines as the length of the fiber is increased due to the power loss of the laser pulse [3]. In our experiment, pre-calibration could not be done before installation, therefore we suggest a post-calibration method that is within the acceptable range of error, ± 0.3 °C. 

**Figure 6:**
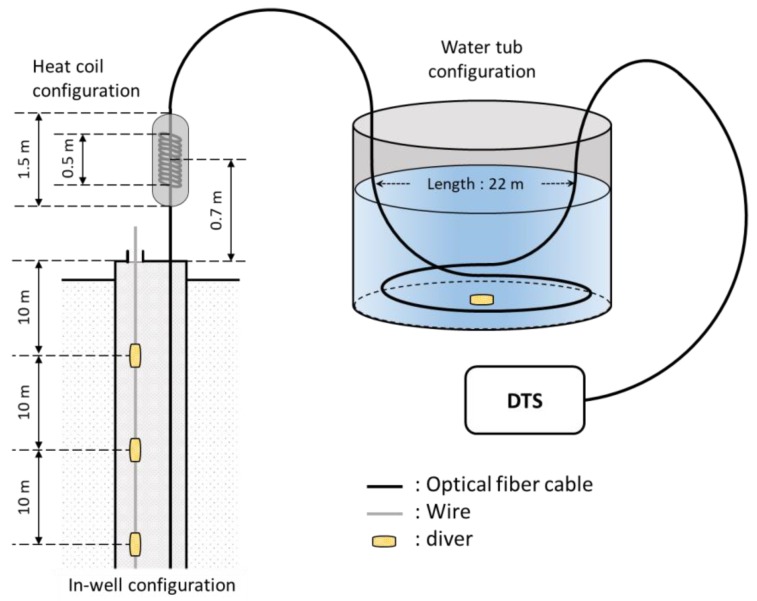
Schematic diagram and setting of three temperature calibration experiments.

Three calibration methods were investigated in the JG-M borehole in April 2018 and the experimental setting is displayed in Figure 6. In a dual-ended configuration, only one absolute temperature point is needed because knowing the temperature at a point results in having two points in this configuration. The “water tub” is the conventional method of correction by submerging about one or more pulse width lengths of fiber cable into water to find the stable absolute temperature. However, submerging enough length of fiber longer than a pulse width length into water may not be possible once the cable is installed. Therefore, two pointwise temperature correction methods are investigated. The ‘heat coil’ heats a portion of fiber cable near the well with a heat coil. The “in-well” is another pointwise method of correction by using the position of a heat coil to apply a slope correction and using the absolute temperature value inside the well to apply the offset correction. For all cases, the slope correction was applied to temperature profile with no events in the well, and background temperature data was collected for 15 min before the calibration method experiments.

#### 3.2.1. Long Point Calibration Method with Mater Tub

**Figure 7:**
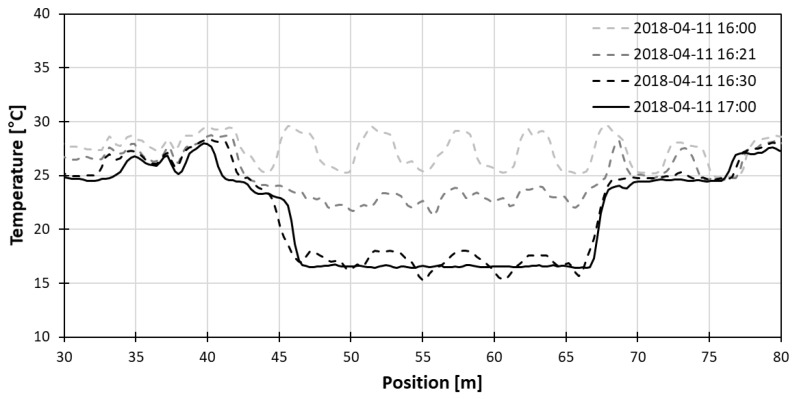
Temperature change with time at the fiber optic cable submerged in water.

In the water tub experiment, 22 m of fiber optic cable, which is longer than a pulse width length of a laser, was submerged in water. Figure 7 shows the process of the temperature being stabilized in the calibration bath. The temperature fluctuation became smaller as time went by, and about 1 h later, finally stabilized to that of water inside the calibration bath.

The position of the cable submerged in water could be clearly seen both on the left and right side of the DTS data, due to the dual-ended installation of the fiber. The stable range on the left side of the data ranges from 48.896 m to 64.148 m, and the stable range on the right side of the data, though it is not displayed in the figure, ranges from 2329.826 m to 2345.078 m. Since the submerged part of the fiber optic cable must have the same temperature, the inner 60 points were averaged to eliminate fluctuation of the temperature value on each side. The midpoint on the left side was at 56.522 m, and midpoint on the right side was at 2337.706 m. To make sure the right midpoint matched the left midpoint, same number of 4887 points to max peak were used.

**Figure 8:**
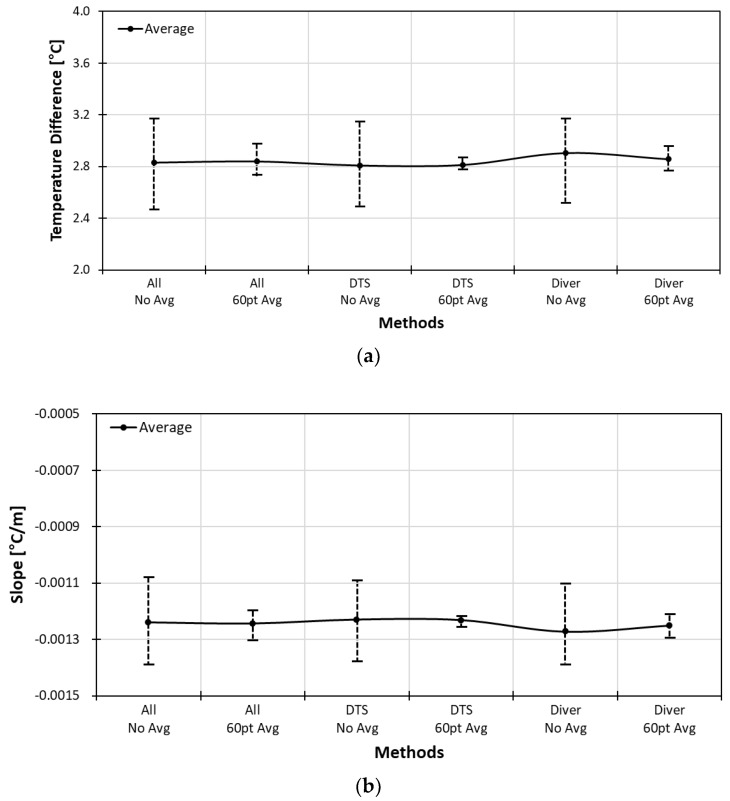
Comparison of long point water tub methods in terms of temperature difference (**a**) and slope range (**b**).

In order to find a better slope correction factor, the temperature change of water within an hour was considered. Even though the specific heat of water is lower than air, the minimum difference of average temperature between the left side and the right side was considered for better estimation of the reference temperature. The ten-minute range which gave the minimum difference between the left and the right side is from 17:53 to 18:02. From the best ten-minute range, the minimum, maximum, and average slope value was used to find the slope equation. The maximum slope gave the minimum difference between the left and the right value. The minimum slope gave the maximum difference between the left and the right value. The slope curve of minimum, maximum and average slope is shown in Figure 8a. In the figure, “all” represents using all the data points without considering the temperature change of water, “DTS” represents the best ten-minute data in terms of minimum difference between the left and the right endpoints. The “Diver” represents the most stable temperature measured with the diver in the water tub for ten minutes.

**Figure 9:**
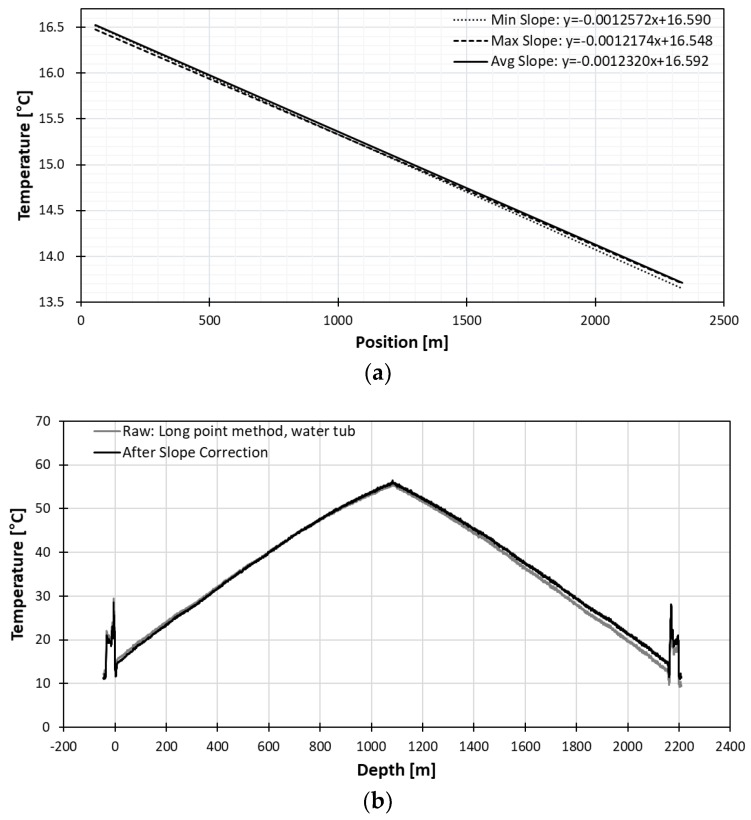
(**a**) Slope curve of long point method with best 10-minute range of DTS 60-point average from 17:53 to 18:02 and (**b**) heat profile after long point water tub temperature correction.

The “DTS” method gave the best result when 60 points were averaged to eliminate fluctuations of the reference temperature as shown in Figure 9a. This was confirmed again by comparing all three methods as displayed in Figure 8. The averaging of 60 points was the most effective in all three cases as it had smaller slope and difference range. 

For all three cases, the slope equation was applied to the background data from April 12 before any experiment because it is best to apply to the data without any events. The temperature accuracy improved after temperature correction, as the difference to true temperature decreased from 2.6 °C before correction to 0.02 °C, as illustrated in Figure 9b.

#### 3.2.2. Pointwise Calibration Method with Heat Coil

The heat-coil was used to locate the position on the DTS data for slope correction. A heat coil of 0.5 m in length was wrapped around the fiber optic cable near the well and insulated with a sponge tube of 1.5 m and wrapped in tape to prevent the temperature from fluctuating due to the wind as shown in Figure 6. The distance from the middle of heat coil to the well was 70 cm. In the heat coil experiment, obtaining a stabilized absolute temperature was difficult because the heat coil was shorter than the pulse width of interrogation system and air temperature varied too quickly. Therefore, the ambient temperature value at the heat coil position was used for slope correction. On April 12, the fiber optic cable was heated with heat coil to about 50 °C for about 10 minutes and the cooling observed for 27 min. 

**Figure 10:**
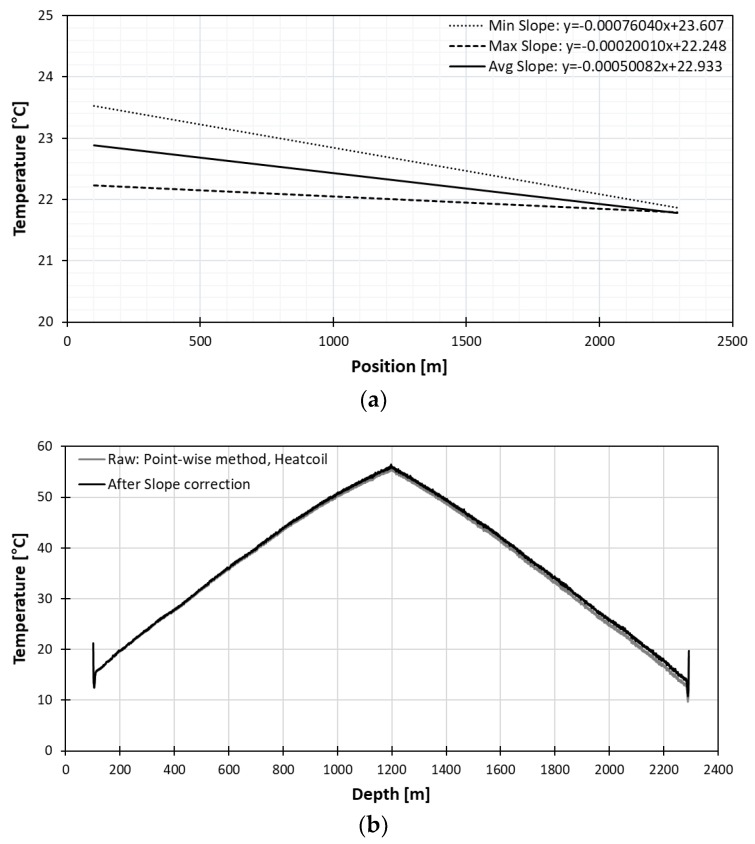
(**a**) Slope curve of pointwise method with heat coil and (**b**) heat profile after pointwise heat coil slope correction.

The slope curves are displayed in Figure 10a, which shows very different slope difference from the long point water tub method. In this case, only slope correction was applied because the absolute temperature was not determined. 

Even with only slope correction, we could evaluate if the calibration is done correctly by comparing the two endpoints because two endpoints on the left and the right should have the same temperature value after correction. However, we can identify in Figure 10b, that temperature at the right endpoint, even with an increase of 1.1 °C after correction, still has a 1.5 °C difference with that of the left endpoint.

#### 3.2.3. In-Well Pointwise Calibration Method 

To compensate the difficulty of obtaining a stable absolute temperature with the heat coil method, three divers were lowered into the well at 10 m, 20 m, 30 m below groundwater which has lower specific heat constant than the air. The diver position on the DTS could be located by counting from the known heat-coil position. The position could also be confirmed by temperature change when the fiber optic meets groundwater, which was only 1 m to in-well water from the heat-coil position.

**Figure 11:**
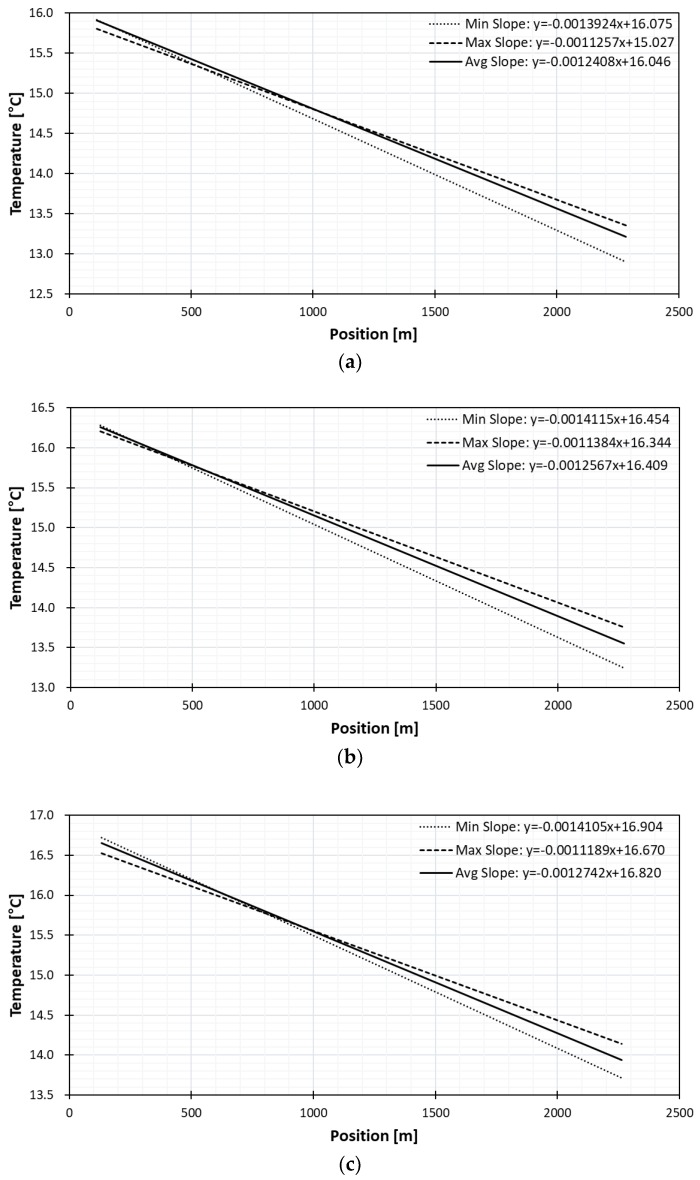
Slope curves of pointwise calibration method by in-well diver at (**a**) 10 m, (**b**) 20 m, (**c**) 30 m.

**Figure 12:**
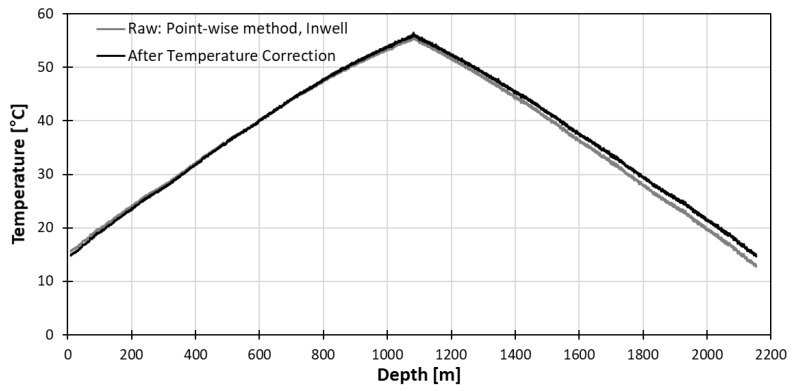
Temperature profile after correction by pointwise method with 10 m in-well diver.

The slopes found with the pointwise in-well method are presented in Figure 11. Compared to the long point water tub method, the variation ranges are wider than the long point method but the average slopes are similar to that of the long point method. The slope and its range did not vary greatly with the depth of diver. The temperature after correction with pointwise in-well diver at 10 m is presented in Figure 12. The temperature accuracy improved as the difference to true temperature is only 0.06 °C compared to the difference of 2.8 °C before correction.

#### 3.2.4. Discussion

**Figure 13:**
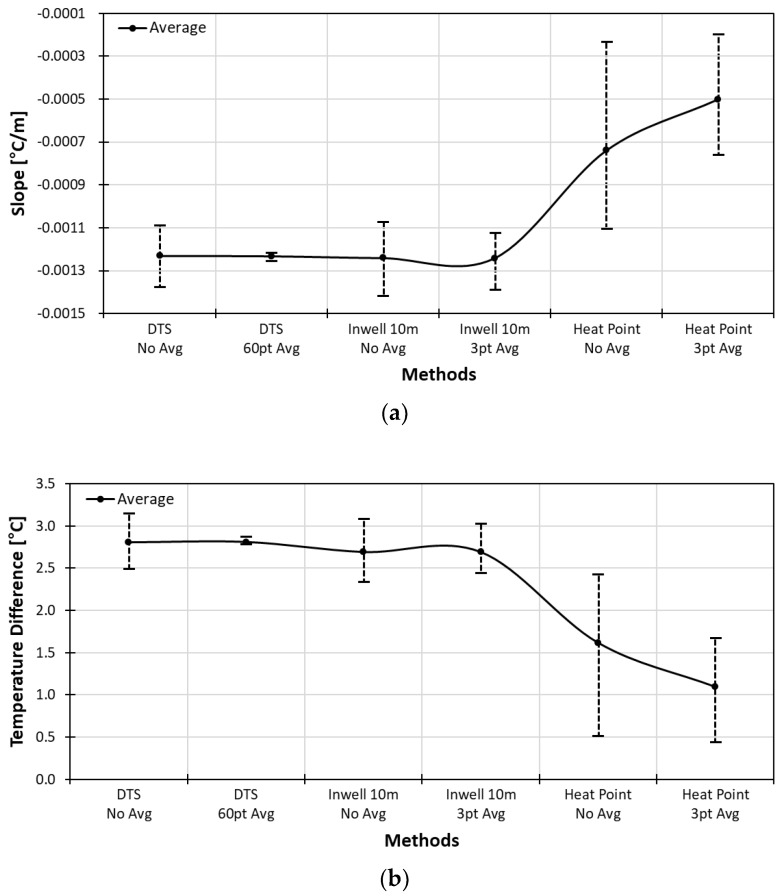
Slope curves (**a**) and temperature difference (**b**) of post-calibration method.

The slope and temperature difference curves of three calibration methods are summarized in Figure 13. The long point calibration method with water tub gave the best result. Although the pointwise calibration method using the diver showed a larger range than the long point method, it gave an average slope and the average temperature difference comparable with that of the long point method. Comparing the variation range of the heat coil method and the in-well method, we can conclude that it is crucial to obtain accurate and stable absolute temperatures. 

The stability analysis was carried by dividing the total round-trip length of the fiber into 10 sections in terms of the same number of sample points per section. The downward trip sections, from wellhead to well bottom, are marked as L5–L1 and the upward trip sections are marked as R1–R5. The number of samples per each section is 897 points for the long point method, 855 points for the pointwise method with in-well diver, and 862 points the pointwise method with heat coil.

**Figure 14:**
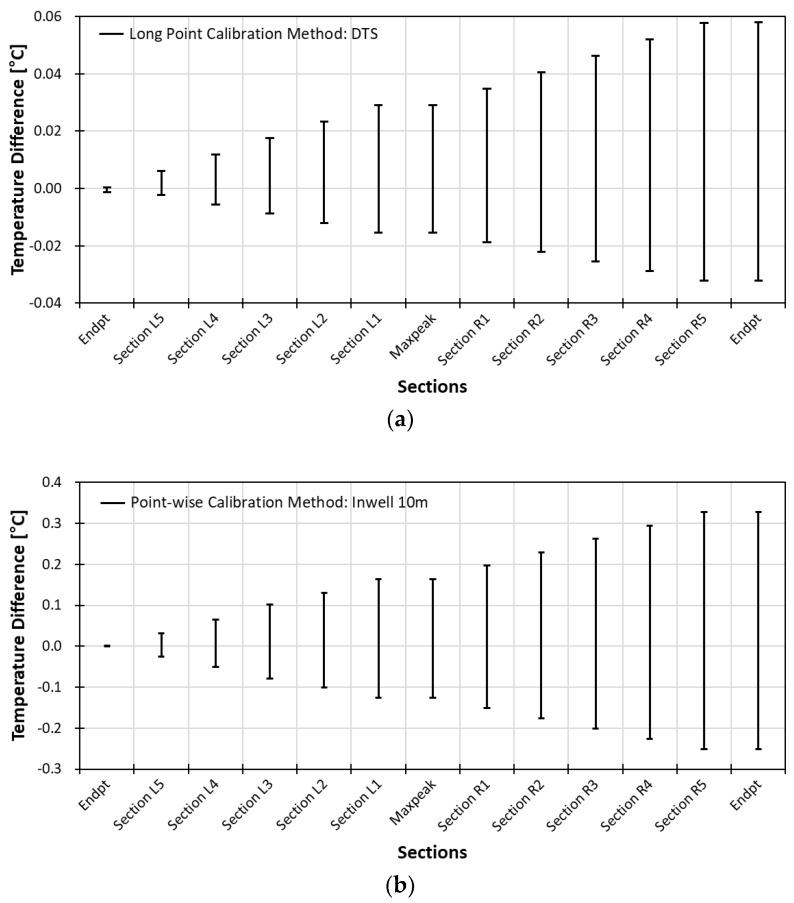

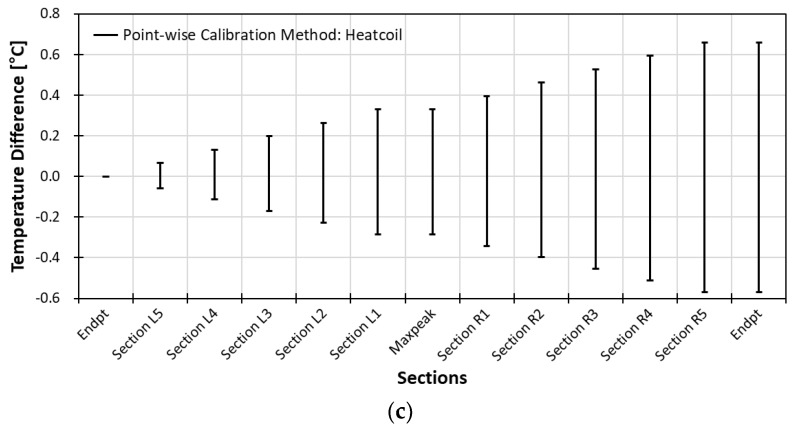
Slope stability of (**a**) long point calibration with water tub (**b**) pointwise calibration with in-well and (**c**) pointwise calibration with heat coil.

The stability curve of all three calibration methods is as shown in Figure 14, which displays the stabilities at 13 points including two top points (Endpt), a bottom point (Maxpeak), and ten midpoints of each section. The slope stability curve of long point calibration method has a range of +0.06 °C and −0.03 °C. The slope stability curve of pointwise calibration method with in-well of 10 m has a range of +0.35 °C and −0.25 °C. The slope stability curve of pointwise calibration method with heat coil has a range of +0.7 °C and −0.6 °C. The temperature difference increases as the length of fiber increase in all three calibration methods as expected due to the power loss that increases as the traveling distance become longer.

The appropriate temperature error range is within 0.3 °C, and both water tub and in-well data fall within the range. However, the error range with water tub slope correction is within 0.3 °C with stability of 0.05 °C and the temperature error range with diver slope correction is within 0.1 °C with a stability of 0.3 °C. Therefore, even though the long point calibration works best, the pointwise calibration method can be a useful alternative where long point calibration cannot be done.

## 4. DTS Monitoring of Well Completion Processes

**Figure 15:**
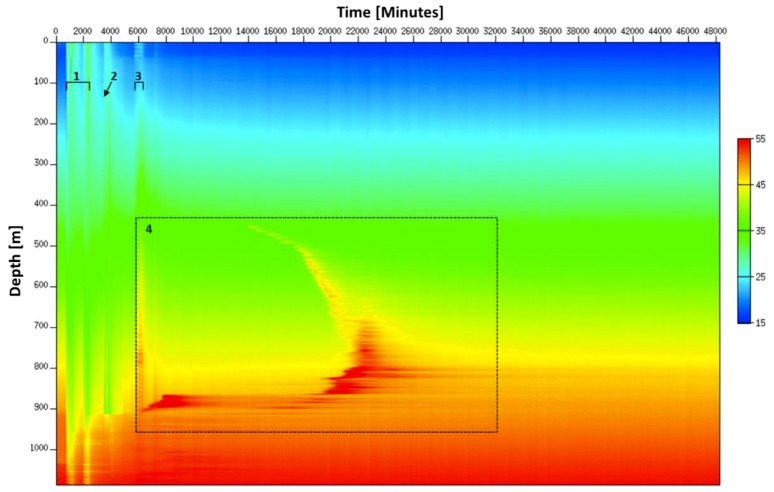
Heat map obtained during well completion where the gravel packing is denoted by 1, the cement injection by 2, the gas lift operation by 3 and the cement curing process by 4.

The uniqueness of the Janggi field DTS data is the continuous real-time monitoring of the whole well completion process from gravel packing to complete curing without well intervention. The heat map obtained from May 11th to June 4th is shown in Figure 15. Anomalies numbered from 1 to 4 correspond to gravel packing, cement injection, gas lift, and cement curing, respectively.

### 4.1. Gravel Packing

**Figure 16:**
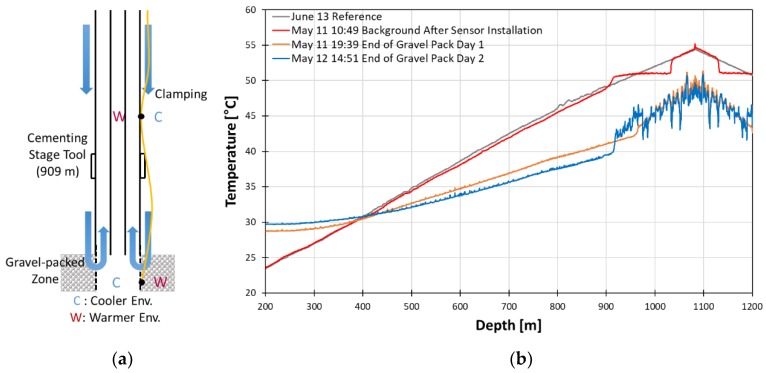
Schematic of gravel packing operation (**a**) and temperature profile during the gravel packing operation (**b**).

There has been monitoring of production in gravel packed completions but monitoring the gravel packing process itself was not yet done [7]. At the Janggi field site, fiber optic cable was connected directly after casing installation to the DTS unit, which allowed real-time monitoring of the gravel packing process throughout the entire length of the well. The detailed temperature profile during gravel packing can be seen in Figure 16 which outlines the major events during the gravel packing process. 

The reference (black line) refers to the temperature profile at the end of well completion. The gravel packing process can be characterized by the decrease of temperature below the reference line. The temperature decreases because of the cooling effect due to the injection of sand mixed with fluid that is cooler than the borehole fluid inside the well. In the deeper parts of the well, high temperature anomalies with oscillating pattern can be seen due to water circulating but leaving sand to be accumulated. The sand deposited in the annulus plays a role of thermal bridge between the surrounding formations and fiber cable on tubing. Because sand has higher heat conductivity than water, such a thermal bridging effect explains the high temperature anomalies. The section where sand is not accumulated does not have this connectivity between formations and cable still shows a low temperature trend. The point where the temperature clearly drops indicate the top level of the gravel pack. The progression up of this anomaly along the well shown in the figure (May 11 19:39 and May 12 14:51) clearly shows that the top of gravel pack can be monitored well by DTS. After gravel packing, the top of gravel pack estimated from DTS data (May 12) matches closely with the planned depth of 910 m as mentioned in Section 3.2.1. 

In the low temperature anomaly section, small peaks that seem like noise are identified. However, they are anomalies due to the clamps being spaced at approximately every 9 m. The clamps provide more direct connection between inside and outside of tubing wall. The effect of clamps appears as high temperature anomalies in the unpacked section while it appears as low temperature anomalies in the gravel-packing zone. In the case of the high temperature anomalies, inside the casing is warmer than the annulus because the sand was directly injected into the annulus. Consequently, the clamped point of the DTS cable is directly connected to the warmer environment while the other parts are not. In the case of low temperature anomalies, inside the casing is cooler in the packed region. Thus, the clamping point has the reverse effect compared to the previous case. The anomalies associated with clamps indirectly shows high resolution of DTS and its usefulness in dynamic condition. 

From the heat map in Figure 15, we can identify that two gravel packing processes that were carried out over the two days have similar patterns to each other. The temperature is increasing above the gravel-packed zone and decreasing in the unpacked zone during the operation. The short burst of cooling at the top may be explained due to either flow change or colder fluid injection or breaks during gravel packing operation. 

The ability to monitor the progression of sand volume allows the possibility to control the amount of sand volume to reach the top of the intended depth and the capability to check for possible plugging as the sand is poured. All these factors help optimize the gravel packing process at the field level as the change in temperature is displayed without interrupting the process. The gravel packing process also showed potential for detecting sand production and the response to sand production can be optimized to diminish the risk of reduced well productivity.

### 4.2. Cement Injection and Curing Process

**Figure 17:**
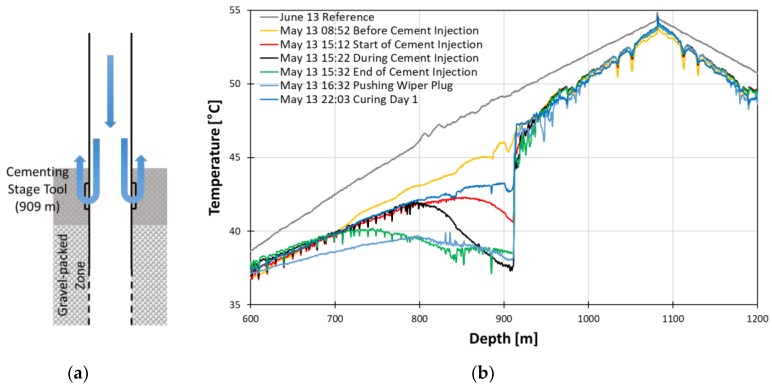
Schematics of cementing injection process (**a**) and temperature profiles during the cement injection process (**b**).

After the gravel packing operation, the cement slurry was injected to the casing to complete the well. The temperature profile of the cement injection process is displayed in Figure 17. The temperature before cement injection has a lower temperature than the reference, which indicates the effect from gravel packing operation still remains and needs more time to recover to an undisturbed normal geothermal gradient. As the slurry is injected, the temperature is being gradually lowered until the end of the injection because the cement slurry is cooler than the borehole fluid. The top of the gravel pack and the bottom of cement can be determined as mentioned in Section 3.2.1.

**Figure 18:**
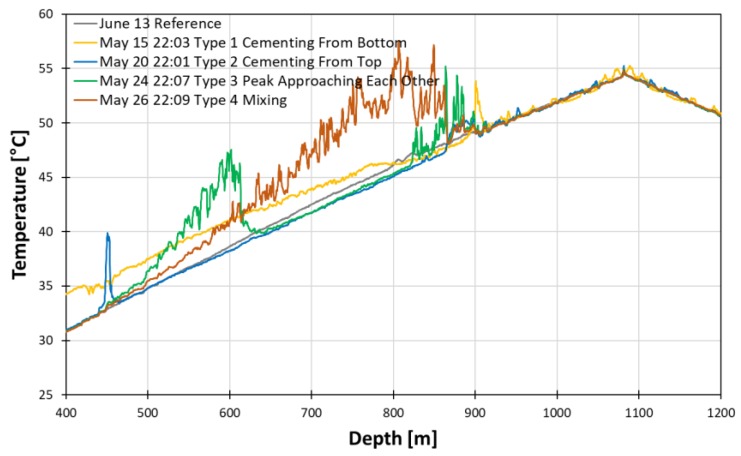
Temperature profiles during the curing process in detail.

The major events during curing process from right after injection to a month after injection are shown in Figure 18. 

In the yellow line denoted as type 1, though the high temperature anomaly due to gas lifting remains at depths shallower than 850 m, we can identify the peak near the stage cement tool which can be interpreted as the start of the exothermic process of the curing. The effect of the gas lift anomaly is gone in type 2 blue line; however, another peak appears near 450 m. The peak indicates the start of curing from the upper part. The type 3 green line displays two peaks from the top and bottom boundary. These boundaries move toward each other, indicating the curing process propagates from the boundary to the intermediate. The type 4 red line displays the two peaks finally meet and start settling down.

From the heat map in Figure 15, the major trends of the whole cementing operation can be observed. We can identify when and where the curing starts, when the curing finally done, and how long it took for complete curing from the heat map provide by constant DTS monitoring. 

The cementing is the final critical steps to ensure the integrity of the well. Therefore, monitoring the entire process from cement slurry injection to curing in real-time can provide key information in order to control and optimizes the process. For example, the progression of injection outlines both injection rate and volume of cement, which offers the ability to check for possible plugging during injection. The constant monitoring of the entire well also allows correlating unusual anomaly to exothermic reaction due to the settling of the cement, which can provide better insights to the different stages of exothermic reactions. As the time of complete curing could be accurately determined by the homogeneity in temperature, monitoring curing process help move on to the next stage with confidence. 

### 4.3. Gas Lift

**Figure 19:**
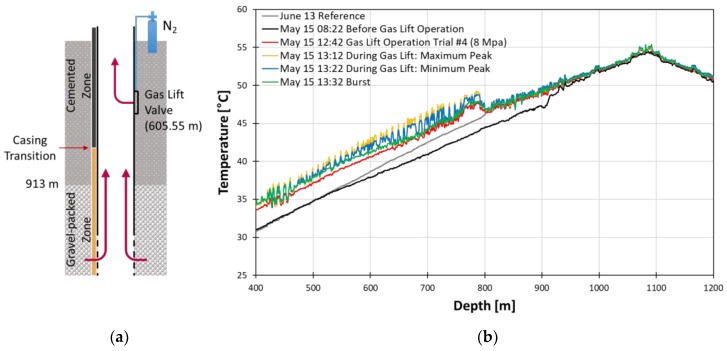
Schematics of gas lift process (**a**) and temperature profiles during the gas lift in May (**b**).

The gas lift process was carried out after cement injection but during cement curing. During the gas lift, the N_2_ gas was released, and the fluid was pulled upwards due to pressure. The major events of one cycle of gas lift operation is displayed in Figure 19. The temperature increased during gas lift operation in the data below 791.90 m. The increase of temperature is due to pulling up of the deeper and warmer reservoir fluid. The high temperature anomaly associated with gas lift operation has a highly oscillating pattern. In our case, however, its amplitude is different at two regions above and below 791.90 m. The difference in amplitude of the oscillation is due to heat conductivity difference of the casing materials. The 791.90 m is the transition point of the two casing materials from steel to FRP. Since steel has higher heat conductivity than FRP, we can observe a higher amplitude of oscillation in the steel casing region than the FRP casing region.

**Figure 20:**
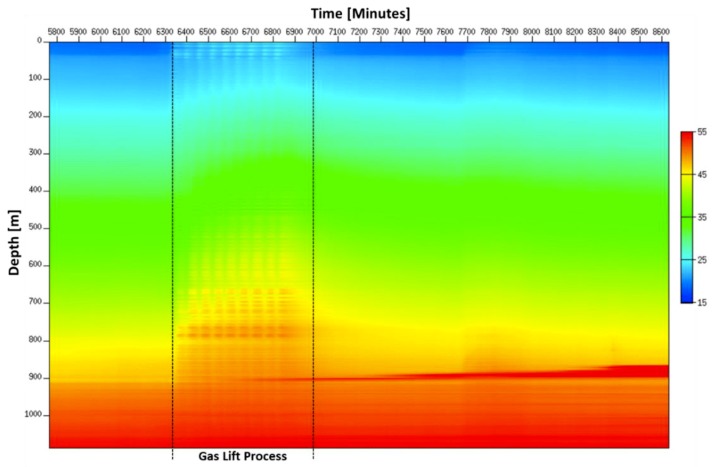
Heat map of entire gas lift operation in May

The heat map during the gas lift cycles which shows the global trend during the gas lift is displayed in Figure 20. The number of repeated patterns during gas lift matches the number of gas lift cycles, which was eight cycles. The width of each repeated pattern is the duration of each cycle. Even after the gas lift operation was finished, its effect lasted a day to return to temperature before the gas lift operation began. The temperature did not completely recover due to the starting of curing process near 900 m during the middle of gas lift operation.

As mentioned in Section 3.1, our gas lift data shows not only a typical highly oscillating thermal anomaly pattern of gas lifting event but also the effect of casing transition, which supports the confirmation of the accuracy of depth correction. The DTS data has the potential to detect multiple points of injection and determine gas rate through each injection point. All these are possible as the event is happening and require no shut-in when measuring DTS temperature data [8]. Therefore, DTS helps capture the temperature response throughout the entire length of the well simultaneously as the event is happening.

## 5. Conclusions

We have presented continuous real-time DTS monitoring data of the whole well completion process from gravel packing to complete curing without well intervention, and discussed the observed thermal anomaly patterns during each well completion process.

The real-time DTS monitoring data revealed that each well completion process can be characterized by its own distinctive temperature anomaly patterns. In addition, it is remarkable that the upward progression of sand and cement slurry and the thermal anomaly due to curing of cement can be monitored. This clearly shows that pre-calibrated DTS, due to its continuous real-time and high spatial resolution nature compared to conventional point sensors and wireline tools, can be a powerful tool not only for monitoring, but also for controlling and optimizing well completion processes without intervention in each process. However, because the thermal event characteristics associated with each well completion process are site- and condition-dependent, further studies to quantify the temperature anomaly depending on factors like injection rate, hydraulic and thermal properties of the formation and fluid etc. are required for quantitative control and optimization of well completion process by DTS. 

For depth calibration, the conventional method distributing the error to the total length is enough. The accuracy could be enhanced more if more points where the depth are known are available in addition to the top and the bottom of well. In the case of temperature calibration, pre-calibration using the conventional method is best. However, our investigation shows that the pointwise method can be an alternative with acceptable accuracy of less than 0.3 °C and stability less than 0.3 °C.
